# Supplementary figures and images for: Genotyping‐by‐sequencing illuminates high levels of divergence among sympatric forms of coregonines in the Laurentian Great Lakes
Source: Evol Appl. 2020 Feb 27;13(5):1037–54. doi: 10.1111/eva.12919 (PMC7232772; doi:10.1111/eva.12919)

K=4

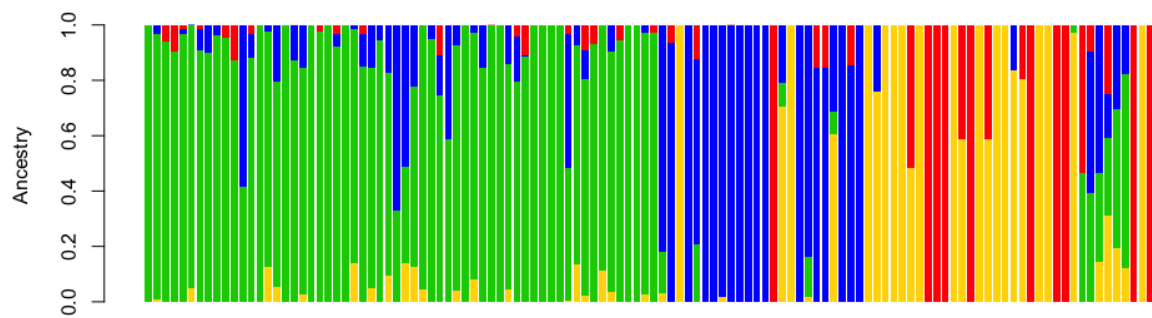

K=5

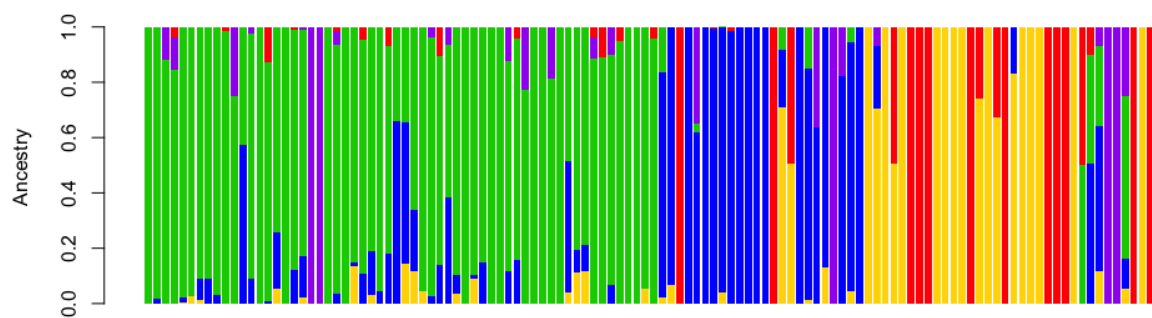

Supplement: Supplementary file 3 [file EVA-13-1037-s003.pdf]

# Generation 0

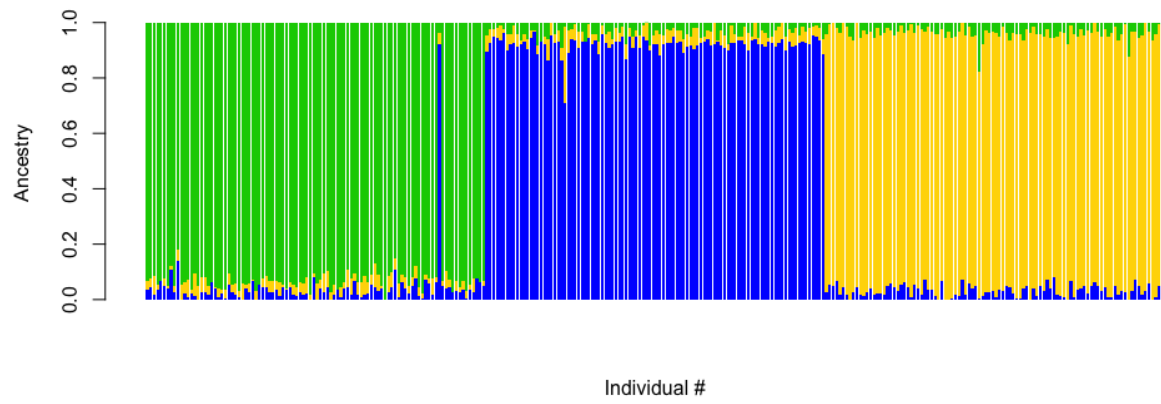

Supplement: Supplementary file 5 [file EVA-13-1037-s005.pdf]

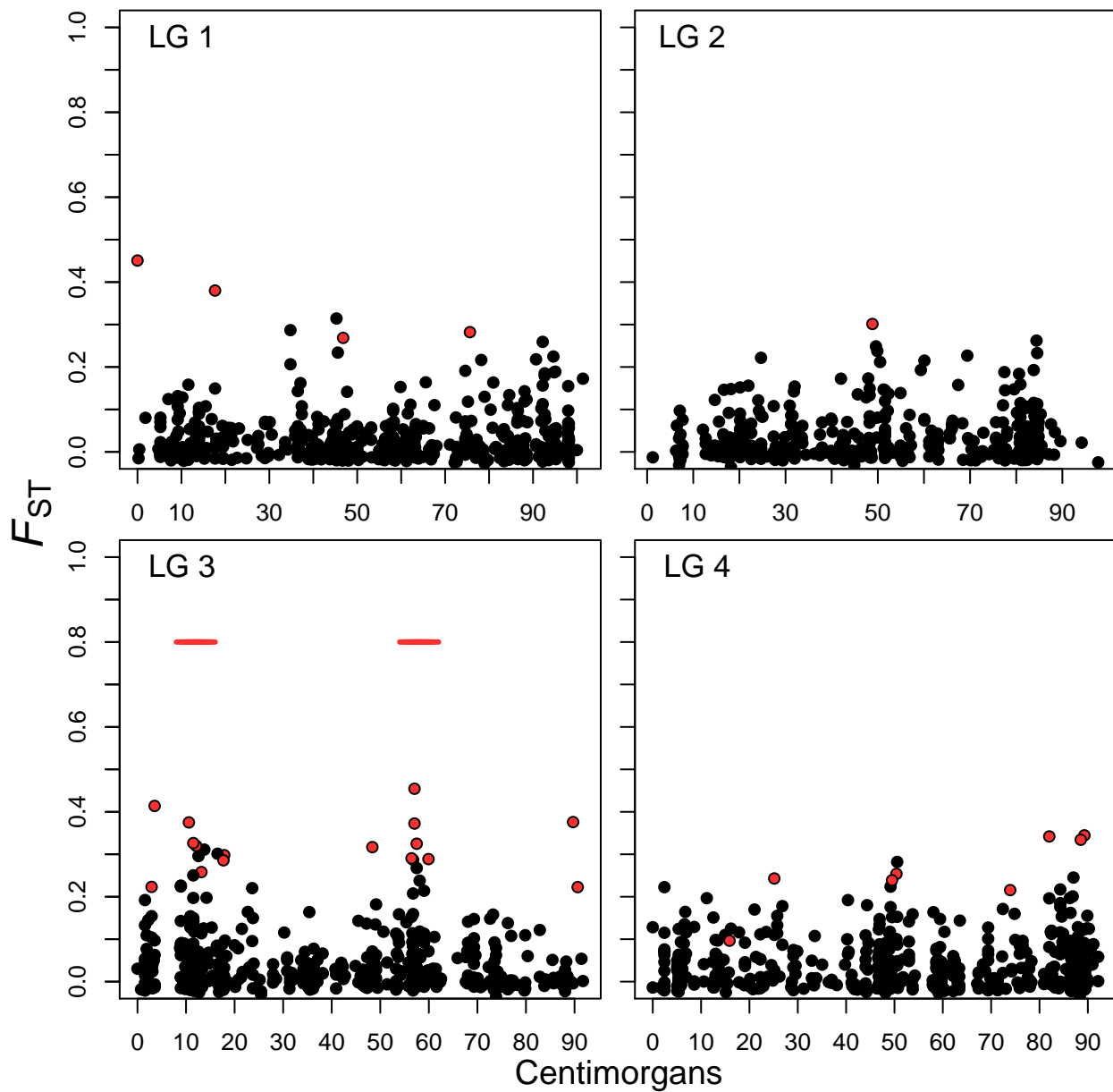

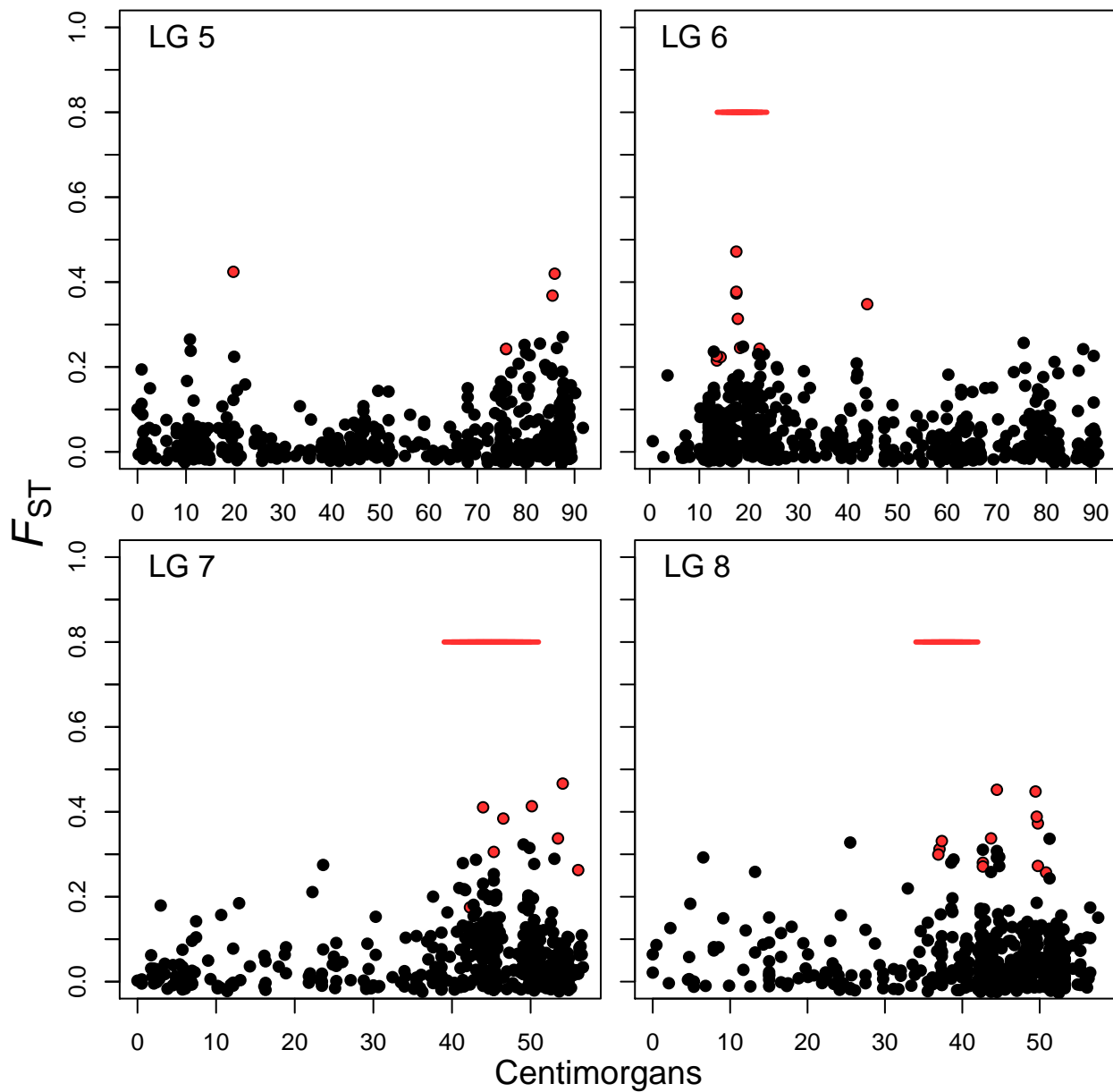

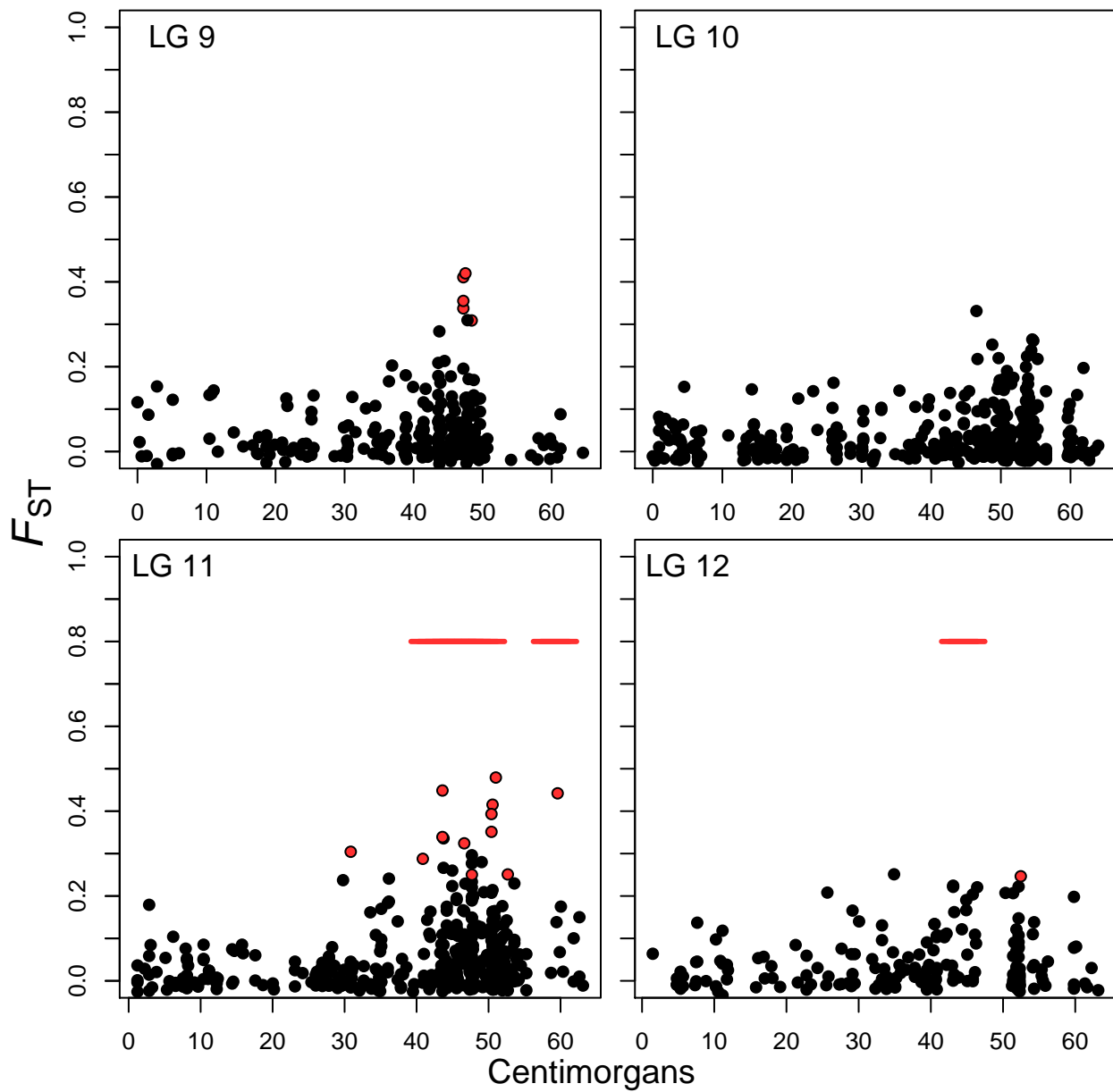

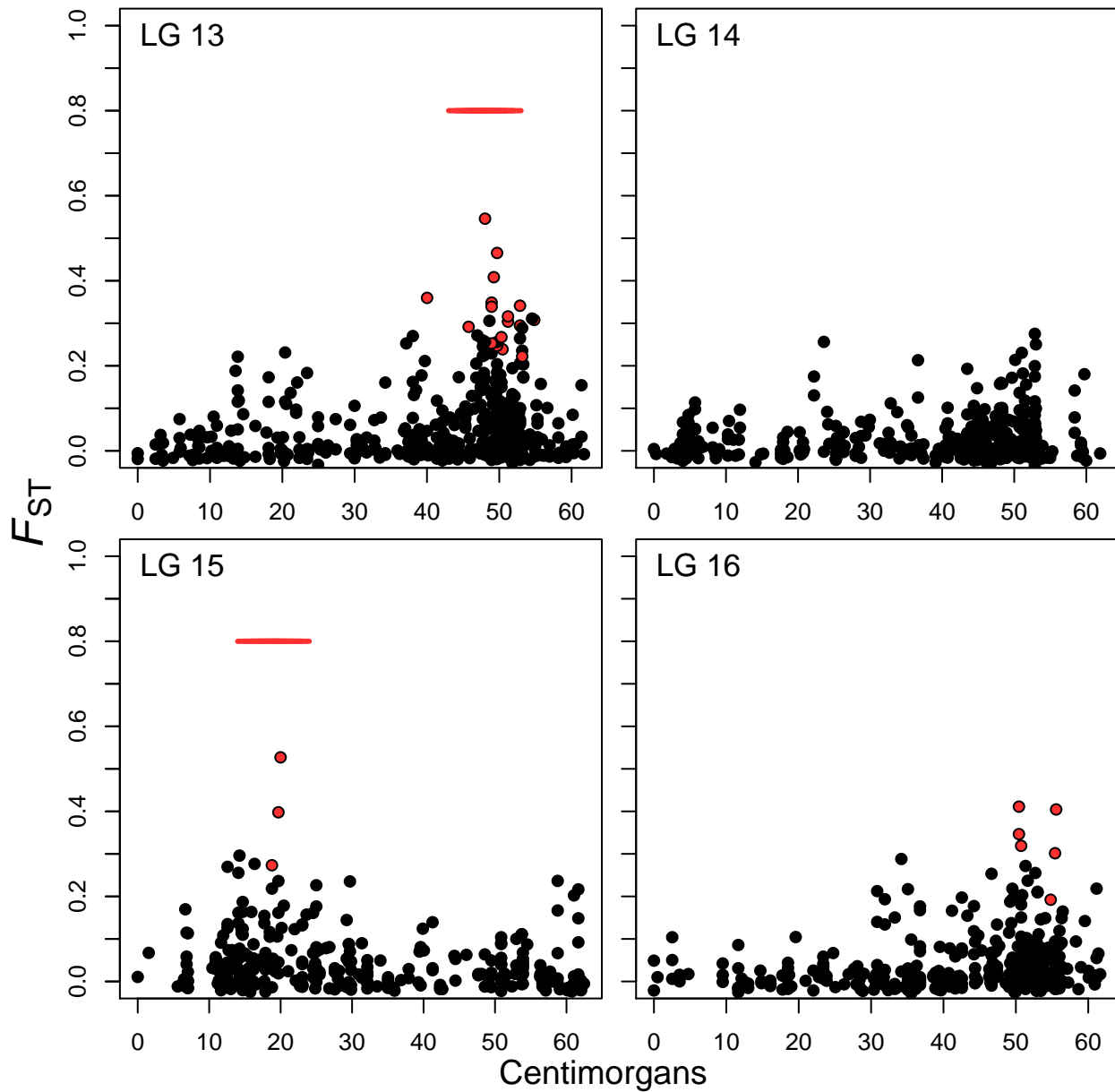

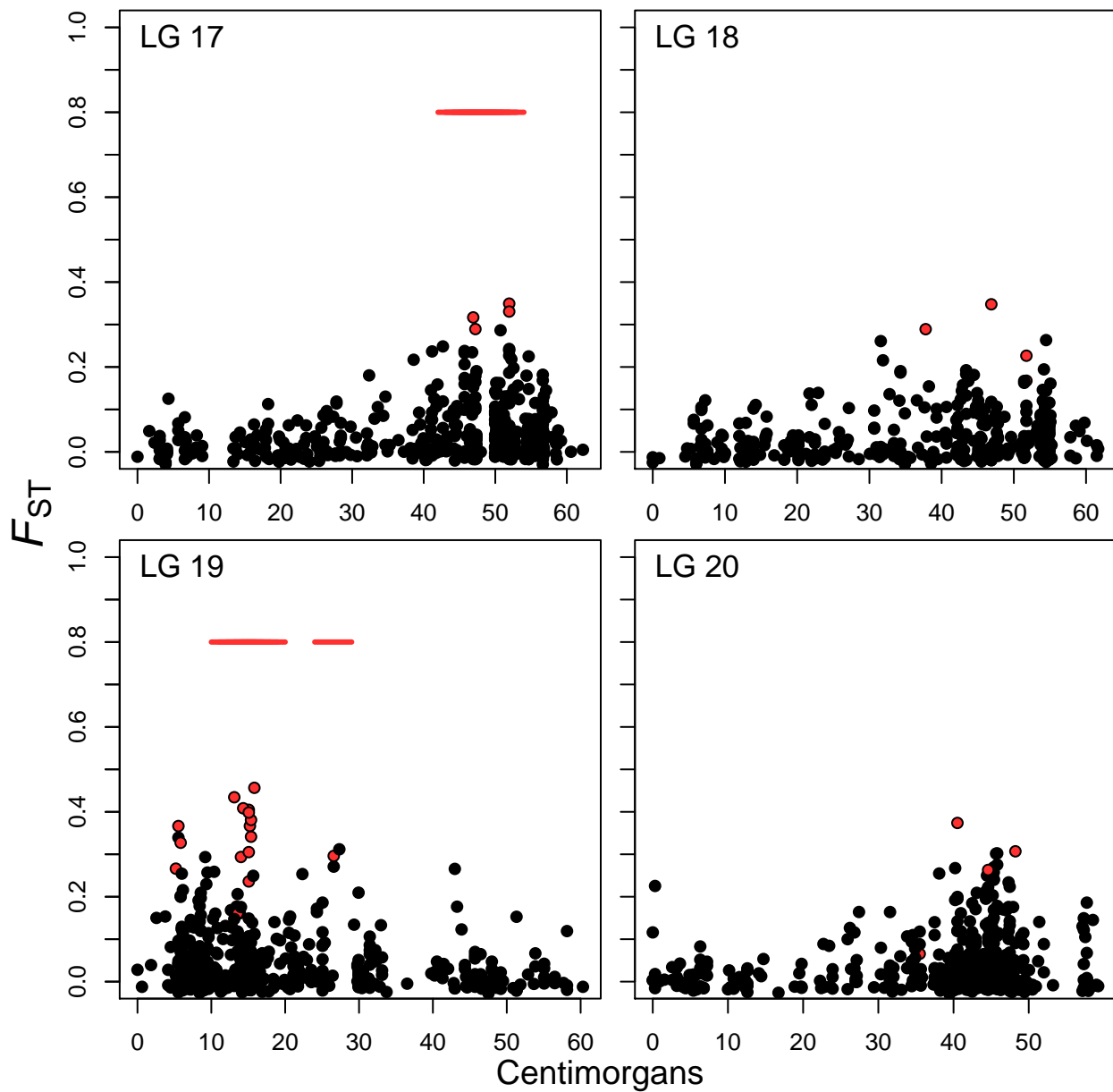

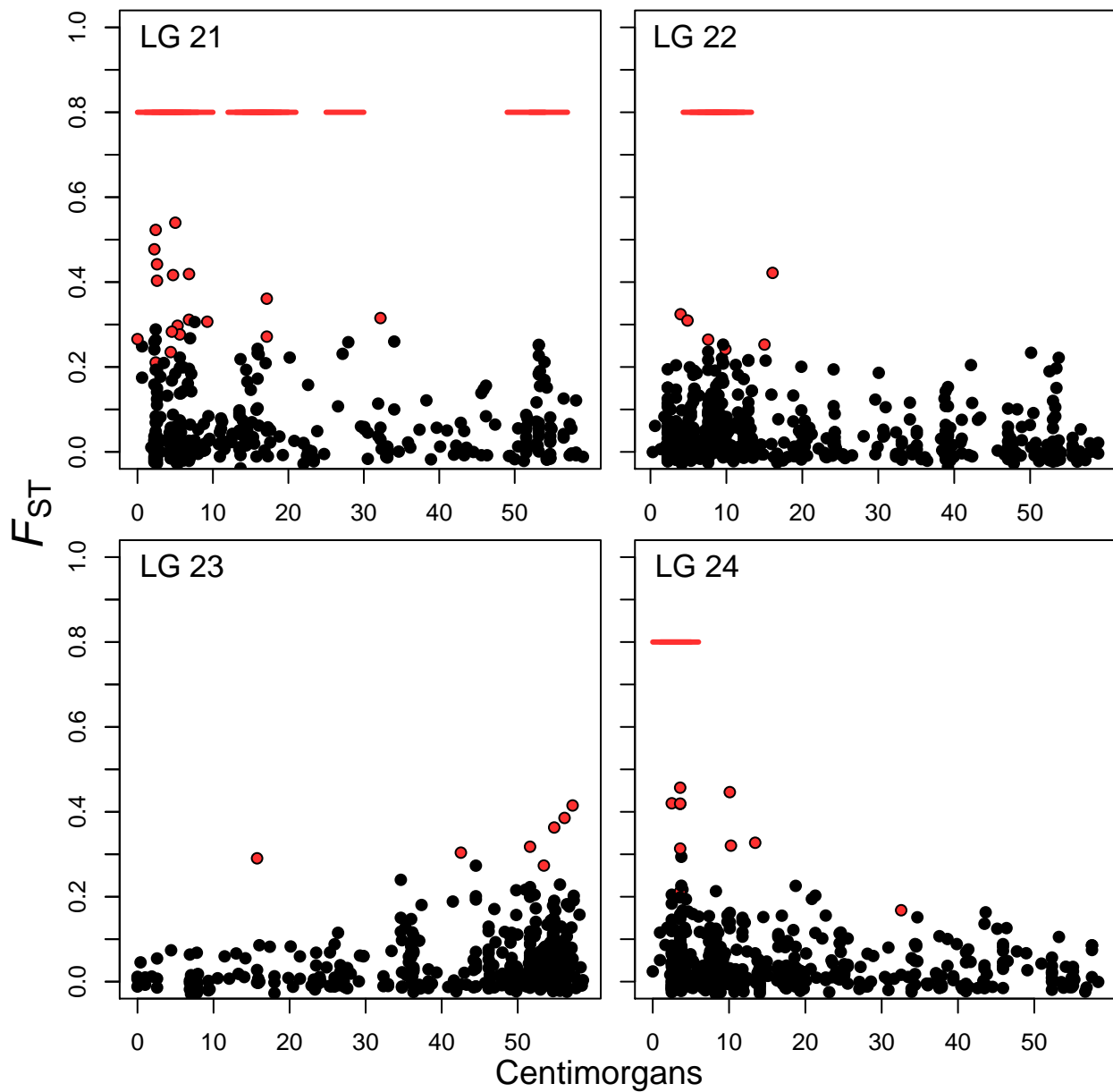

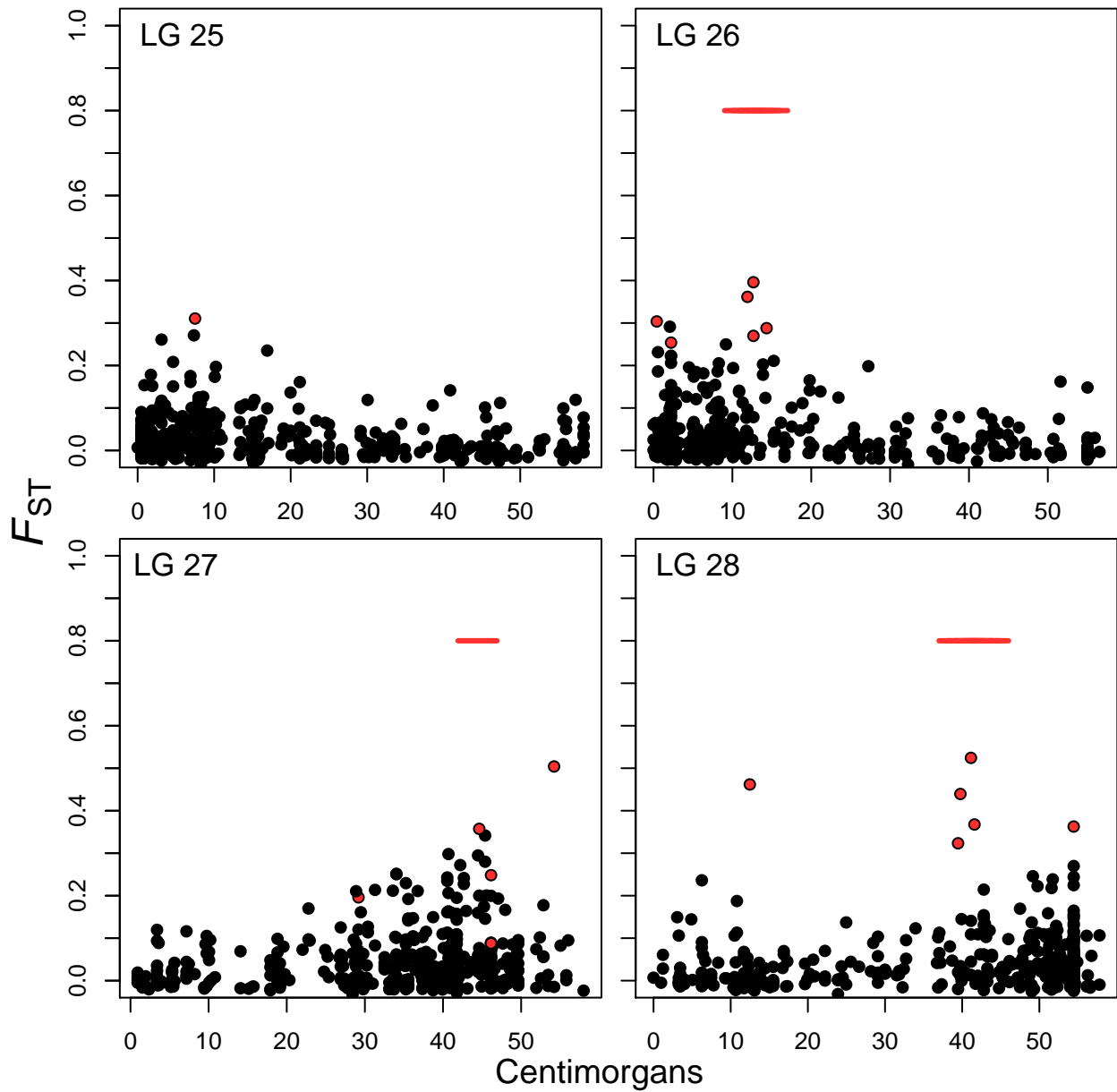

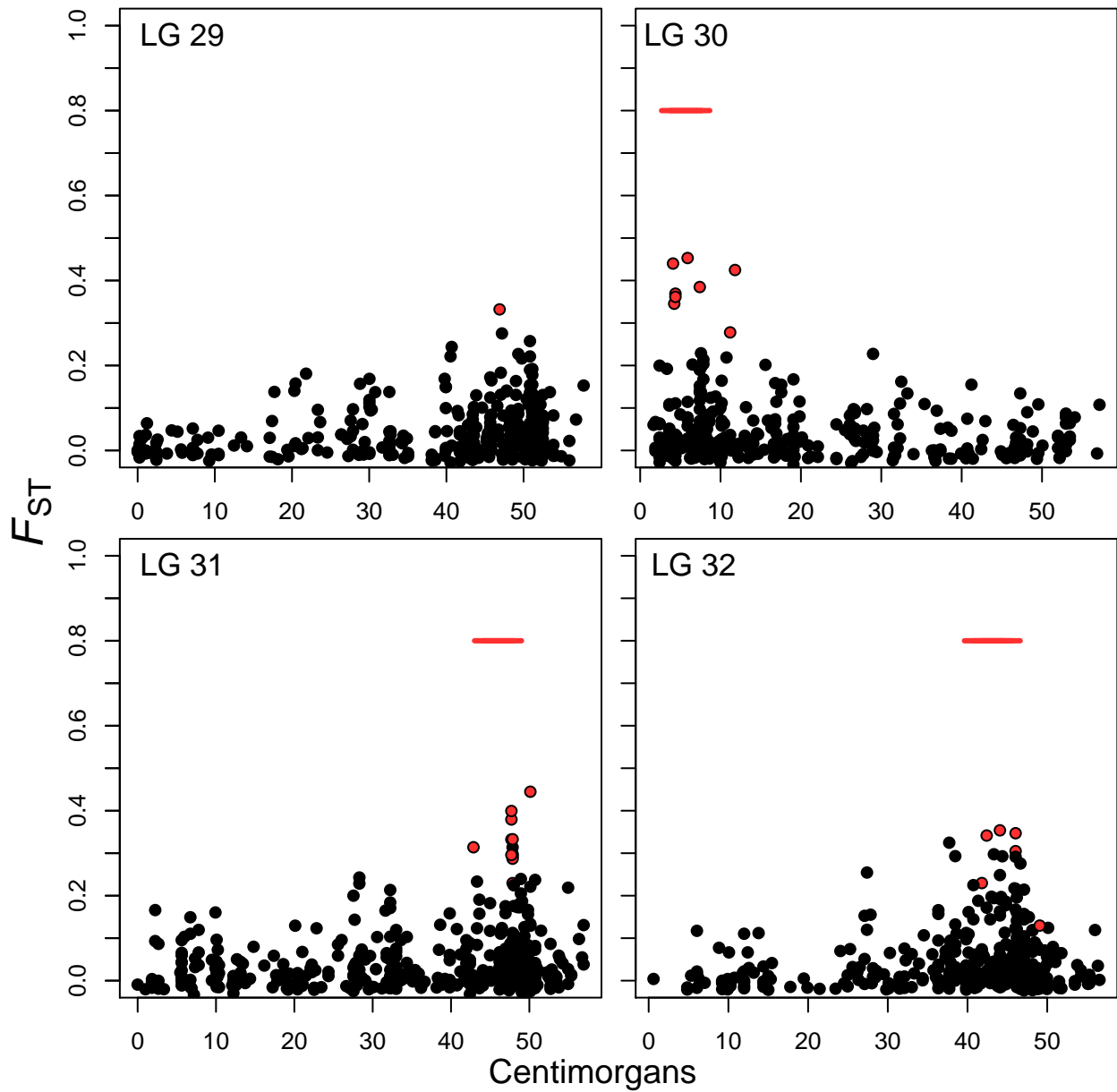

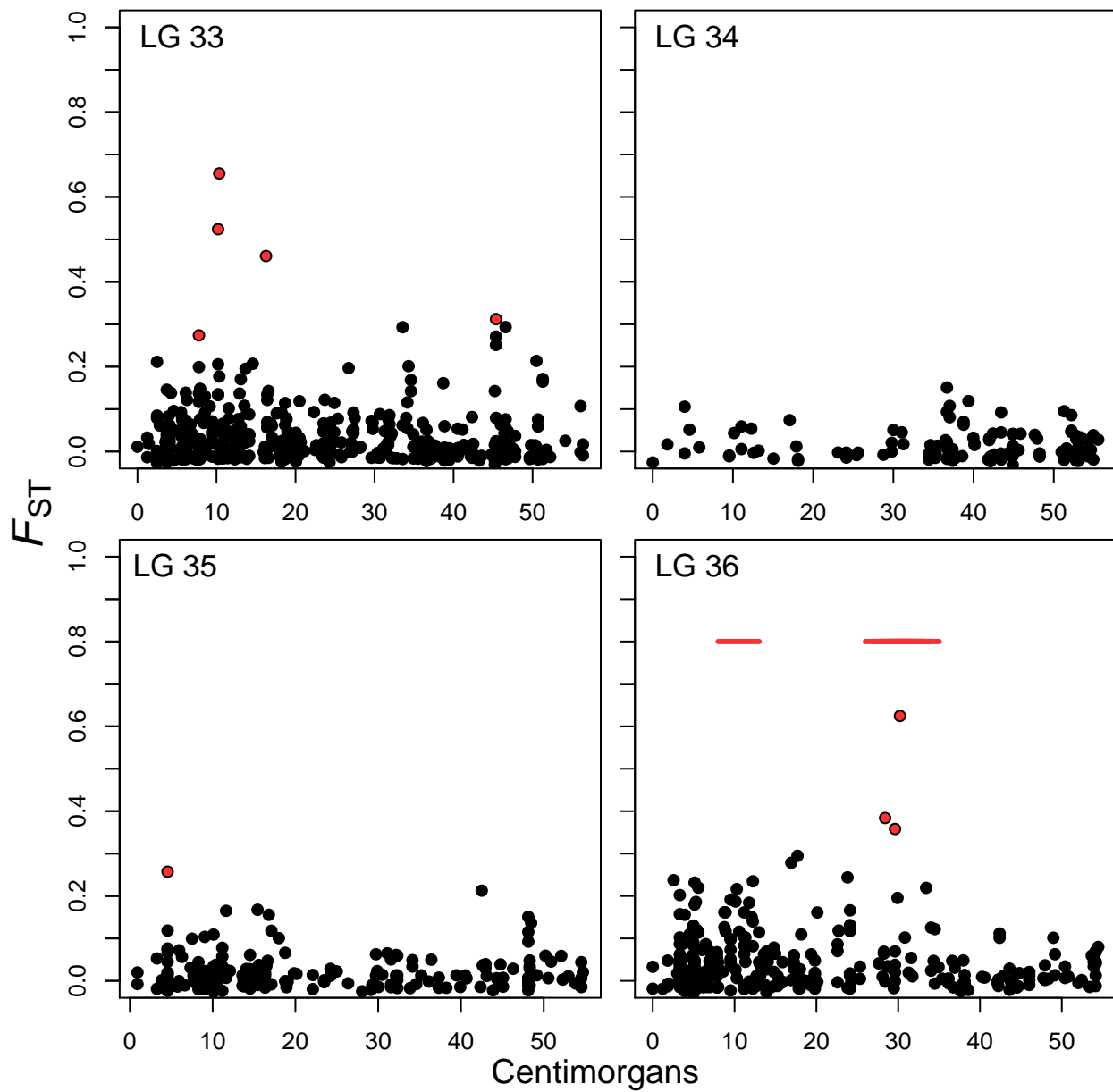

$F_{ST}$ 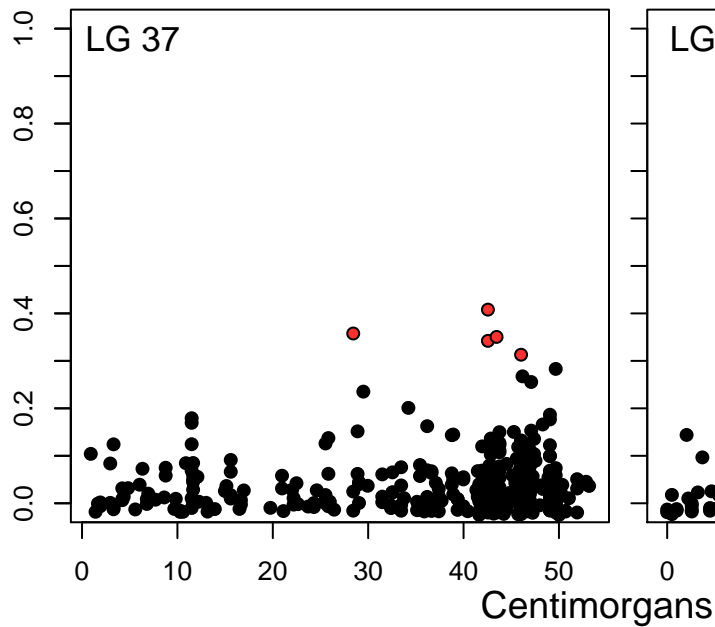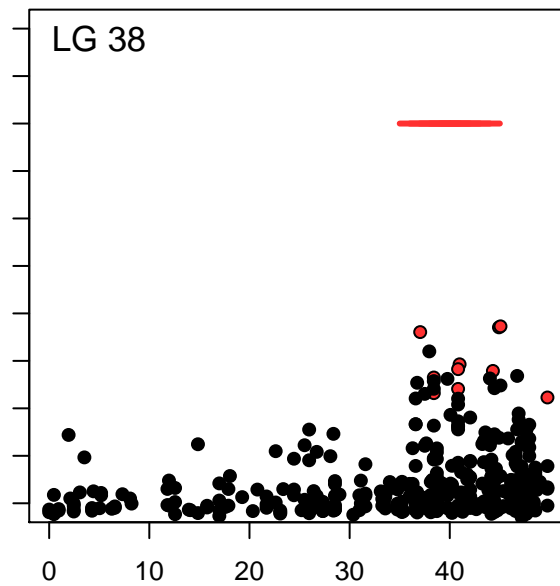

Supplement: Supplementary file 6 [file EVA-13-1037-s006.pdf]

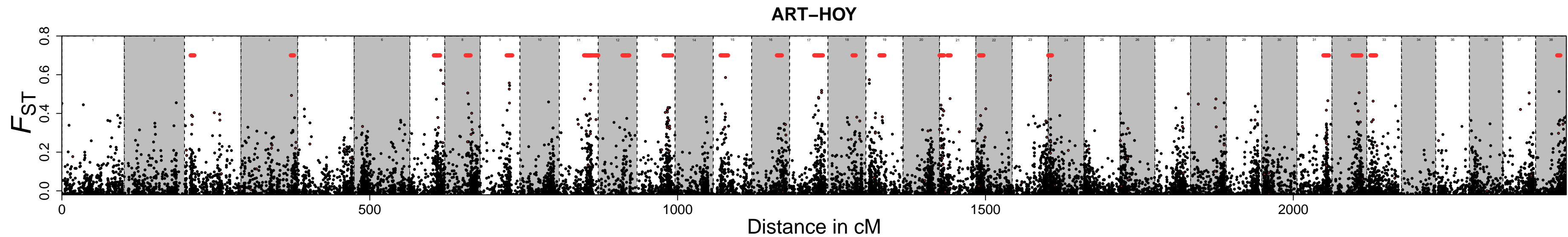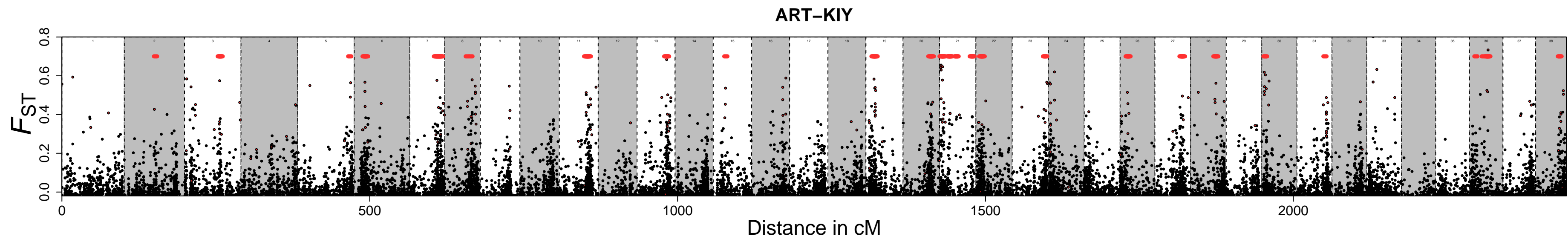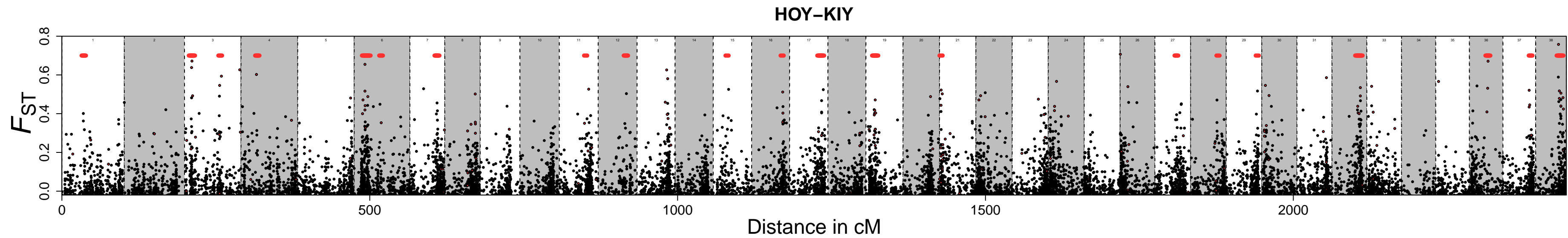

Supplement: Supplementary file 7 [file EVA-13-1037-s007.pdf]
